# Dietary acid load and markers of malnutrition, inflammation, and oxidative stress in hemodialysis patients

**DOI:** 10.3389/fnut.2024.1369206

**Published:** 2024-03-22

**Authors:** Arghavan Balali, Marilyn S. Nehls, Hadi Tabibi, Atefeh As’habi, Arman Arab

**Affiliations:** ^1^Student Research Committee, Isfahan University of Medical Sciences, Isfahan, Iran; ^2^Department of Community Nutrition, School of Nutrition and Food Science, Nutrition and Food Security Research Center, Isfahan University of Medical Sciences, Isfahan, Iran; ^3^Department of Kinesiology and Health Promotion, University of Kentucky, Lexington, KY, United States; ^4^Department of Clinical Nutrition & Dietetics, Faculty of Nutrition and Food Technology, National Nutrition and Food Technology Research Institute, Shahid Beheshti University of Medical Sciences, Tehran, Iran; ^5^Food Safety Research Center (Salt), Semnan University of Medical Sciences, Semnan, Iran; ^6^Division of Sleep Medicine, Harvard Medical School, Boston, MA, United States; ^7^Medical Chronobiology Program, Division of Sleep and Circadian Disorders, Departments of Medicine and Neurology, Brigham and Women's Hospital, Boston, MA, United States

**Keywords:** acid load, nutrition, hemodialysis, malnutrition, inflammation

## Abstract

**Aims:**

The present study was conducted to examine the association between dietary acid load (DAL) and markers of inflammation, oxidative stress, and malnutrition in a group of Iranian hemodialysis (HD) patients.

**Methods:**

This cross-sectional study was performed on individuals aged ≥18 years who were on HD at least 6 months before their enrollment in the study. A 4-day dietary recall was used for the evaluation of dietary intake. DAL was calculated using two methods including potential renal acid load (PRAL) and net endogenous acid production (NEAP). For assessing the malnutrition status, we used the subjective global assessment (SGA), dialysis malnutrition score (DMS), and malnutrition inflammation score (MIS). Fasting blood samples were collected from each participant to assess serum levels of high-sensitivity C-reactive protein (hs-CRP), soluble intercellular adhesion molecule-1 (sICAM-1), soluble vascular adhesion molecule-1 (sVCAM-1), sE-selectin, malondialdehyde (MDA), nitric oxide (NO), and endothelin-1.

**Results:**

In total, 291 patients with a mean age of 57.73 ± 0.88 years and HD vintage of 4.27 ± 0.25 months were enrolled in the current study. Significant positive associations were observed between PRAL and hs-CRP (*β* = 1.77, 95% CI: 0.88, 2.65), sICAM-1 (*β* = 83.21, 95% CI: 10.39, 156.04), sVCAM-1 (*β* = 194.63, 95% CI: 74.68, 314.58), and sE-selectin (*β* = 6.66, 95% CI: 1.81, 11.50) among participants with the highest PRAL scores, compared to those with the lowest PRAL scores. NEAP was positively correlated with hs-CRP (*β* = 1.34, 95% CI: 0.46, 2.22), sICAM-1 (*β* = 88.83, 95% CI: 16.99, 160.67), and MDA (*β* = 0.35, 95% CI: 0.005, 0.71). Additionally, marginally significant higher odds of SGA (OR = 1.98, 95% CI: 0.95, 4.11) and DMS (OR = 1.94, 95% CI: 0.92, 4.05) were observed in individuals in the third tertile of PRAL vs. the first tertile of PRAL. NEAP had also a marginally significant positive correlation with DMS (OR = 2.01, 95% CI: 0.93, 4.31).

**Conclusion:**

This study illustrates that higher consumption of acidic foods is correlated with markers of inflammation, oxidative stress, and malnutrition in HD patients.

## Introduction

End-stage renal disease (ESRD) is a major public health concern with a global prevalence of 11–13% of the population (1, 2). The ESRD incidence rate has been increasing in recent years, and it is associated with a significant economic burden for the affected individuals and healthcare systems (1). A high prevalence of protein-energy malnutrition (PEM) and inflammation have been observed in ESRD patients undergoing hemodialysis (HD) (3). PEM and inflammation are believed to be intertwined and are correlated with high morbidity, cardiovascular mortality, and all-cause mortality in maintenance HD patients (3). The strong association between PEM and inflammation has given rise to the medical phenomena known as the malnutrition inflammation atherosclerosis (MIA) syndrome and malnutrition-inflammation complex syndrome (MICS) (3). Thus, it is of great importance to seek useful strategies for the management of malnutrition and inflammation in HD patients.

Possible causes of MICS include but not limited to comorbid illnesses, uremia, low clearance of inflammatory factors, anorexia, and loss of soluble nutrients during HD (4). Moreover, researchers believe that diet may be a significant contributor to MICS (5). Previous studies indicate that the intake of acidic foods, particularly animal proteins, in individuals whose endogenous acid–base balance is disturbed was associated with metabolic acidosis and subsequent malnutrition and inflammation (6). To explore the contribution of acidic dietary compounds to various health markers, dietary acid load (DAL) has been defined to quantify the acidic potential of the diet.

In epidemiological studies, two methods have been used for the evaluation of DAL including potential renal acid load (PRAL) (7) and net endogenous acid production (NEAP) (8). Both PRAL and NEAP are validated approaches for assessing DAL in renal transplant recipients due to these methods having a significant correlation with 24-h urinary net acid excretion (NAE) (9). Therefore, they are speculated to be useful indicators of DAL status in patients with HD.

Previous studies have found a correlation between DAL and diseases such as diabetic nephropathy (10), kidney stones (11), and kidney cancer (12). However, to our knowledge, no study has previously investigated the predictive role of DAL in HD patients. Therefore, in the present cross-sectional study, we intended to investigate the association between DAL and markers of inflammation, oxidative stress, and malnutrition in HD patients among a sample of Iranian adults. We anticipated that higher DAL is associated with greater inflammation and oxidative stress levels and unfavorable malnutrition status in HD patients.

## Methods

### Study population and design

In the current cross-sectional study, 2,302 HD patients were selected from August 2019 to June 2020 from multiple HD centers in Tehran, Iran. To identify appropriate participants, the list of all the HD centers in Tehran was obtained from the Iran Dialysis Center, where the names of all the HD patients were taken from each of the 50 HD centers, and then the names of the patients who met the eligibility criteria to be enrolled in this study were recorded (*n* = 2,302). Next, the names of HD centers in Tehran were sorted alphabetically, and then the names of the patients in these centers were listed. Finally, 291 out of 2,302 subjects were selected using the systematic sampling method. Prior to enrollment in the study, written consent was obtained from all patients. The present study was conducted based on the Declaration of Helsinki, and the study protocol was also approved by the Ethics Committee of the National Nutrition and Food Technology Research Institute of Iran (IR.SBMU.NNFTRI.REC.1387.319). HD patients aged ≥18 years who were on hemodialysis at least 6 months prior to enrollment were included in the study. However, patients with a history of liver disease, inflammatory diseases, malignancies, chronic or acute pancreatitis, and HIV infection were excluded. All patients underwent HD three times a week for 4 h using polysulfone capillary dialyzers and bicarbonate dialysate.

### Assessment of dietary intakes

The dietary intakes of participants were evaluated by an expert dietitian using a 4-day, 24-h dietary recall, including 2 dialysis days and 2 non-dialysis days through face-to-face interviews. For the examination of the daily intakes of energy, macronutrients, and micronutrients, Nutritionist IV software (First Databank, Hearst Corp., San Bruno, CA, United States), modified for Iranian foods, was used. Since dietary intakes of patients may be different on dialysis vs. non-dialysis days, both days were selected to capture day-to-day variations in diet (13). Participants were asked to recall all the drinks and food items consumed within 24 h. Portion size models were used to help people estimate portion size and improve accuracy.

### DAL calculation

DAL was calculated using 2 surrogate measures, PRAL and NEAP, which have both been routinely used in epidemiological studies. The PRAL score was calculated using the following equation by Remer et al. (7): PRAL (mEq/d) = [0.49 × protein intake (g/d) + 0.037× dietary phosphorous (mg/d) – 0.021 × dietary potassium (mg/d) – 0.013 × calcium (mg/d) – 0.026 × magnesium (mg/d)]. Moreover, the NEAP score was estimated using the following algorithm of Frasseto et al. (8): NEAP (mEq/d) = [54.4 × protein intake (g/d) ÷ potassium intake (mEq/d) − 10.2]. Negative values of PRAL and NEAP indicate an alkaline potential of a diet; however, positive PRAL and NEAP scores illustrate an acidic potential of a diet (12, 14).

We used both PRAL and NEAP scores for acid load assessment due to their differences in nutritional aspects and biological mechanisms. In the PRAL method, the intestinal absorption rates of protein, potassium, calcium, magnesium, and phosphate were considered, and this method has been validated against urine pH in healthy populations. Moreover, the NEAP score considers sulfuric acid generation rate from the metabolism of protein and bicarbonate production from the metabolism of potassium salts. In addition, a previous study found a positive correlation between PRAL and NEAP (correlation coefficient = 0.84, *p* < 0.001) (12, 14).

### Assessment of malnutrition

We used the subjective global assessment (SGA), dialysis malnutrition score (DMS), and malnutrition inflammation score (MIS) to determine the malnutrition status of subjects. The SGA, which is a validated tool for the assessment of malnutrition status in HD patients, consists of two criteria: (1) medical history (i.e., dietary intake, functional capacity, weight change, and gastrointestinal symptoms) and (2) clinical assessment (i.e., physician’s grading of the loss of subcutaneous fat, presence of edema, and muscle wasting). Each component had three levels of severity, including normal nutritional status (grade A), mild to moderately affected (grade B), and severely affected (grade C). Accordingly, participants with grades of A, B, and C were placed in the corresponding well-nourished, mild to moderately malnourished, and severely malnourished groups (15, 16). The DMS, a modified quantitative version of SGA, includes 7 variables (i.e., muscle wasting, functional capacity, loss of subcutaneous fat, gastrointestinal symptoms, weight change, dietary intake, and comorbidity) that each score from 1 (normal) to 5 (very severe), resulting in a total score of 7–35 (17). Patients were classified into three categories based on DMS: (1) normal nutrition status (score of 7–13), (2) mild to moderately malnourished (score of 14–23), and (3) severely malnourished (score of 24–35). The MIS was generated by adding the total iron-binding capacity (TIBC) (mg/dL), serum albumin (g/dL), and body mass index (BMI) (kg/m^2^) to DMS, including four levels of severity from 0 (normal) to 3 (severely abnormal). The sum score of MIS ranges from 0 to 30 and is categorized into three groups: 0–7 (well-nourished), 8–18 (mild to moderately malnourished), and 19–30 (severely malnourished) (17).

### Biochemical assessment

The serum concentrations of high-sensitivity C-reactive protein (hs-CRP) were determined using ELISA kits (Diagnostics Biochem Canada, London, Canada) with an intra-and inter-assay CV of 4.6%. The serum levels of soluble intercellular adhesion molecule-1 (sICAM-1), soluble vascular adhesion molecule-1 (sVCAM-1), and sE-selectin were also evaluated by Diaclone ELISA kits (Diaclone, Besancon, France). Intra-and inter-assay CVs for sICAM-1, sVCAM-1, and sE-selectin were 3.5, 6.3, and 6.7%, respectively. Endothelin-1 was evaluated with Biomedica kits (Biomedica, Vienna, Austria) with an intra-and inter-assay CV of 8.5%. The colorimetric method was used to assess serum nitric oxide (NO) and serum malondialdehyde (MDA) concentrations with an intra-and inter-assay CV of 7.8% for NO and 4.6% for MDA (Cayman Chemical, Ann Arbor, MI, United States).

### Assessment of other variables

The measurement of dry weight was conducted with a digital scale (Omron BF511, Omron Corp., Kyoto, Japan) to the nearest 100 g at the end of a dialysis session. For the assessment of height, an upright measuring tape was used, with measurements taken to the nearest 1 mm. To calculate BMI, weight in kg was divided by squared height in meters (m^2^). To evaluate the dialysis adequacy based on the Kt/V index, post-dialysis weight, duration of dialysis, ultrafiltration volume, and pre-and post-dialysis serum urea levels were used (17). Dialysis vintage is presented as the time in months that each patient has spent on HD. Blood samples of 8 milliliters were collected from participants after a 10–12 h fast and immediately centrifuged at 2500 rpm for 15 min to isolate serum. Serum specimens were stored at −70°C for later analyzes. Serum concentrations were measured using a colorimetric method for creatinine and calcium, a photometric method for urea and phosphorous, a Bromocresol green approach for albumin, and a flame photometric approach for serum potassium. All biochemical analyzes were conducted using Pars Azmoon (Tehran, Iran) commercial kits.

### Statistical analysis

All analyzes were conducted using SPSS version 26 (IBM Corp., Armonk, NY, United States). Prior to data analysis, the normality of variables was explored via the skewness statistic, Q-Q plot, and Kolmogorov–Smirnov test. The subjects were first categorized into tertiles according to PRAL and NEAP scores. Tertile categorization was chosen because it is more practical for nutritional interpretation as subjects classify in three qualitative groups (low/intermediate/high). Quantitative and qualitative variables were expressed as mean ± standard deviation (SD) and frequency (percentage), respectively. The difference between continuous variables across tertiles of PRAL and NEAP was assessed via one-way analysis of variance (ANOVA). The distribution of categorical variables across tertiles of PRAL and NEAP was examined using the Chi-squared test. The association between DAL and the biomarkers of oxidative stress and inflammation was investigated using multiple linear regression analysis, and beta (*β*) estimates with 95% confidence intervals (CIs) were reported for three different models. The first model was controlled for age (continuous), sex, and total energy intake (continuous). The next model was additionally adjusted for dialysis adequacy (continuous), dialysis vintage (continuous), serum potassium (continuous), serum calcium (continuous), serum phosphorous (continuous), serum urea (continuous), and albumin (continuous). Further adjustment was made for BMI (continuous) in the last model. Multiple logistic regression analysis was implemented to assess the link between DAL and malnutrition in the three adjusted models, and odds ratios (OR) with corresponding 95% CIs were reported. The first model was adjusted for age (continuous), sex, and total energy intake (continuous). The next model was additionally adjusted for dialysis adequacy (continuous), dialysis vintage (continuous), and serum urea (continuous). The last model was also adjusted for serum potassium (continuous), serum calcium (continuous), and serum phosphorous (continuous). *p*-values <0.05 were considered statistically significant, and all analyzes were two-tailed.

## Results

In total, 291 HD patients, including 127 women and 164 men were included in the current study with a mean (SD) age of 57.73 ± 15.17 years, BMI of 24.11 ± 4.45 kg/m^2^, dialysis vintage of 4.27 ± 4.43 months, and dialysis adequacy of 1.23 ± 0.35 Kt/V. The general characteristics of participants across tertiles of PRAL and NEAP are presented in Table 1. As can be seen, height, serum urea, serum phosphorous, hs-CRP, sICAM-1, sVCAM-1, and sE-selectin were significantly higher in patients with the highest scores of PRAL compared to those with the lowest scores. Moreover, participants in the highest tertile of PRAL were more likely to be male. In terms of NEAP tertiles, the mean serum urea, hs-CRP, and sICAM-1 were significantly higher in patients in the highest tertile of NEAP compared to patients in the lowest tertile of NEAP. No other significant differences were seen across PRAL and NEAP tertiles regarding other variables (Table 1).

**Table 1:** Characteristics of the study population across tertiles of dietary PRAL and NEAP.

Variables	Tertiles of dietary PRAL				Tertiles of dietary NEAP			
	T1 [< 6.94 (mEq/d)]	T2 [6.94 to 13.47 (mEq/d)]	T3 [> 13.47 (mEq/d)]	*p*-value	T1 [< 70.47 (mEq/d)]	T2 [70.47 to 93.23 (mEq/d)]	T3 [> 93.23 (mEq/d)]	*p*-value
*N*	97	97	97		97	97	97	
PRAL	1.44 ± 5.78	10.26 ± 1.85	20.84 ± 6.98	< 0.001	2.52 ± 6.83	11.73 ± 5.84	18.29 ± 8.29	< 0.001
NEAP	60.96 ± 15.29	88.42 ± 21.88	112.44 ± 36.93	< 0.001	57.73 ± 10.53	81.76 ± 6.61	122.32 ± 33.30	< 0.001
*General information*								
Age (years)	58.64 ± 15.25	56.62 ± 14.89	57.93 ± 15.44	0.644	56.82 ± 16.48	57.08 ± 14.44	59.28 ± 14.53	0.466
Sex								
Female	51 (52.6)	43 (44.3)	33 (34.0)	0.009	48 (49.5)	38 (39.2)	41 (42.3)	0.312
Male	46 (47.4)	54 (55.7)	64 (66.0)		49 (50.5)	59 (60.8)	56 (57.7)	
Weight (kg)	64.11 ± 15.02	62.63 ± 12.63	65.35 ± 13.12	0.380	62.49 ± 13.97	65.02 ± 13.04	64.58 ± 13.86	0.388
Height (m)	1.61 ± 0.09	1.62 ± 0.10	1.65 ± 0.09	0.011	1.61 ± 0.09	1.63 ± 0.09	1.63 ± 0.10	0.223
BMI (kg/m^2^)	24.65 ± 5.15	23.79 ± 4.12	23.89 ± 4.00	0.337	23.93 ± 4.68	24.26 ± 4.23	24.14 ± 4.48	0.875
Dialysis adequacy (Kt/V)	1.25 ± 0.37	1.25 ± 0.36	1.19 ± 0.33	0.425	1.28 ± 0.38	1.22 ± 0.34	1.18 ± 0.34	0.190
Dialysis vintage (months)	49.71 ± 51.05	52.12 ± 62.69	51.95 ± 44.90	0.940	52.70 ± 52.13	47.53 ± 52.41	53.56 ± 55.40	0.696
Serum creatinine (mg/dL)	9.40 ± 3.34	8.60 ± 3.18	8.43 ± 3.10	0.084	9.23 ± 3.42	8.72 ± 3.17	8.48 ± 3.06	0.257
Serum urea (mg/dL)	114.80 ± 26.43	126.80 ± 30.56	127.09 ± 29.26	0.004	113.06 ± 26.78	130.61 ± 28.35	125.06 ± 30.05	< 0.001
Serum potassium (mEq/L)	4.45 ± 0.98	4.53 ± 1.07	4.53 ± 0.98	0.809	4.51 ± 0.95	4.47 ± 1.07	4.53 ± 1.01	0.900
Serum calcium (mg/dL)	9.31 ± 0.50	9.19 ± 0.44	9.28 ± 0.48	0.165	9.28 ± 0.47	9.25 ± 0.50	9.26 ± 0.45	0.852
Serum phosphorous (mg/dL)	3.65 ± 0.38	3.62 ± 0.39	3.76 ± 0.34	0.028	3.66 ± 0.41	3.64 ± 0.34	3.73 ± 0.37	0.221
Albumin (g/dL)	4.07 ± 0.37	4.11 ± 0.37	4.17 ± 0.34	0.175	4.07 ± 0.37	4.16 ± 0.32	4.12 ± 0.39	0.239
*Inflammatory biomarkers*								
hs-CRP (mg/L)	4.64 ± 3.42	5.68 ± 3.06	6.31 ± 2.85	0.001	4.96 ± 3.41	5.06 ± 3.25	6.61 ± 2.58	< 0.001
sICAM-1 (ng/mL)	601.57 ± 220.17	679.46 ± 249.19	681.90 ± 271.03	0.039	606.95 ± 210.19	657.51 ± 258.20	698.52 ± 270.37	0.038
sVCAM-1 (ng/mL)	668.11 ± 347.88	847.65 ± 472.39	858.05 ± 417.89	0.002	733.39 ± 422.66	792.15 ± 441.17	848.94 ± 402.87	0.166
sE-selectin (ng/mL)	21.86 ± 13.18	25.58 ± 14.06	27.98 ± 21.12	0.036	23.25 ± 14.07	25.85 ± 13.63	26.35 ± 21.15	0.383
MDA (μmol/L)	5.69 ± 0.71	5.88 ± 1.57	5.82 ± 0.98	0.471	5.66 ± 0.71	5.80 ± 1.29	5.94 ± 1.32	0.236
NO (μmol/L)	42.24 ± 9.80	43.83 ± 10.58	41.49 ± 9.75	0.259	42.30 ± 9.55	43.20 ± 10.85	42.05 ± 9.82	0.706
Endothelin-1 (pg/mL)	26.16 ± 25.47	26.24 ± 26.39	30.47 ± 27.29	0.431	24.94 ± 25.09	27.73 ± 25.35	30.19 ± 28.57	0.386
*Malnutrition indicators*								
SGA								
Normal	41 (42.3)	38 (39.2)	34 (35.1)	0.303	39 (40.2)	45 (46.4)	29 (29.9)	0.141
Malnutrition	56 (57.7)	59 (60.8)	63 (64.9)		58 (59.8)	52 (53.6)	68 (70.1)	
DMS								
Normal	38 (39.6)	38 (39.2)	32 (33.0)	0.344	37 (38.5)	45 (46.4)	26 (26.8)	0.091
Malnutrition	58 (60.4)	59 (60.8)	65 (67.0)		59 (61.5)	52 (53.6)	71 (73.2)	
MIS								
Normal	45 (46.9)	44 (45.4)	41 (42.3)	0.520	44 (45.8)	53 (54.6)	33 (34.0)	0.098
Malnutrition	51 (53.1)	53 (54.6)	56 (57.7)		52 (54.2)	44 (45.4)	64 (66.0)	

Data are presented as mean ± SD or number (% within tertiles of dietary PRAL and NEAP).

p-value obtained from analysis of variance (ANOVA) for continuous variables and Chi-squared test for categorical variables.

BMI, Body mass index, hs-CRP, High-Sensitivity C Reactive Protein; sICAM, Serum Intercellular Adhesion Molecule; sVCAM, Serum Vascular Cell Adhesion Molecule; MDA, Malondialdehyde; NO, Nitric Oxide; LP, Lipoprotein; TG, Triglyceride; TC, Total Cholesterol; HDL, High-Density Lipoprotein; LDL, Low-Density Lipoprotein; SGA, Subjective Global Assessment; DMS, Dialysis Malnutrition Score; MIS, Malnutrition Inflammation Score; PRAL, Potential Renal Acid Load; NEAP, Net Endogenous Acid Production.

The dietary intake of macro- and micro-nutrients of participants across tertiles of PRAL and NEAP are described in Table 2. Patients in the highest tertile of PRAL had significantly higher intakes of total energy, protein, zinc, and vitamins B_2_, B_3_, and B_12_ than those in the lowest tertile of PRAL. Moreover, lower intakes of carbohydrates, total fiber, mono-unsaturated fatty acids (MUFA), magnesium, vitamin C, and folic acid were observed in patients in the third tertile of PRAL than patients in the first tertile of PRAL. Participants in the third tertile of NEAP had significantly higher intakes of total energy, protein, iron, and vitamin B_3_. However, intakes of total fat, total fiber, polyunsaturated fatty acids (PUFA), MUFA, magnesium, vitamin C, and folic acid were significantly lower in patients in the highest tertile of NEAP in comparison to patients in the lowest tertile of NEAP.

**Table 2:** Selected nutrient intake of participants across tertiles of dietary PRAL and NEAP.

Variables	Tertiles of dietary PRAL				Tertiles of dietary NEAP			
	T1	T2	T3	*p*-value	T1	T2	T3	*p*-value
Energy (kcal/day)^b^	1076.74 ± 469.20	1300.91 ± 428.03	1591.88 ± 459.59	< 0.001	1224.10 ± 510.51	1399.87 ± 499.24	1345.55 ± 472.41	0.042
Protein (g/d)	43.53 ± 5.42	49.11 ± 7.98	57.23 ± 10.87	< 0.001	44.00 ± 7.26	50.40 ± 9.65	55.48 ± 9.72	< 0.001
Fat (g/d)	38.75 ± 9.10	36.15 ± 9.44	36.82 ± 11.81	0.183	39.62 ± 9.63	37.92 ± 10.64	34.19 ± 9.68	< 0.001
Carbohydrate (g/d)	202.86 ± 20.16	201.07 ± 21.38	187.36 ± 36.05	< 0.001	200.69 ± 20.85	195.62 ± 26.03	194.91 ± 34.33	0.285
Total fiber (g/d)	8.26 ± 2.26	7.04 ± 1.95	7.02 ± 5.62	0.028	8.46 ± 2.32	7.11 ± 1.94	6.76 ± 5.54	0.003
SFA (g/d)	9.40 ± 2.81	9.09 ± 3.25	9.94 ± 4.54	0.259	9.72 ± 3.08	9.53 ± 3.86	9.18 ± 3.87	0.570
PUFA (g/d)	13.20 ± 4.59	12.36 ± 4.81	11.55 ± 6.21	0.093	13.73 ± 5.10	12.94 ± 5.33	10.45 ± 4.90	< 0.001
MUFA (g/d)	11.49 ± 3.53	10.03 ± 3.43	10.60 ± 4.67	0.035	11.47 ± 3.62	10.95 ± 4.44	9.72 ± 3.58	0.006
Magnesium (mg/d)	112.38 ± 29.32	104.60 ± 22.95	102.28 ± 29.04	0.027	118.69 ± 28.99	109.41 ± 21.06	91.21 ± 24.62	< 0.001
Zinc (mg/d)	4.04 ± 1.04	4.44 ± 1.19	4.95 ± 1.65	< 0.001	4.38 ± 1.18	4.64 ± 1.34	4.43 ± 1.56	0.360
Iron (mg/d)	9.41 ± 1.84	9.75 ± 2.36	10.15 ± 3.34	0.143	9.45 ± 2.09	9.50 ± 1.96	10.35 ± 3.41	0.027
Selenium (μg/d)	0.06 ± 0.01	0.06 ± 0.02	0.11 ± 0.28	0.067	0.06 ± 0.02	0.06 ± 0.02	0.10 ± 0.28	0.166
Vitamin E (mg/d)	2.18 ± 1.64	2.02 ± 2.44	1.82 ± 1.72	0.448	2.26 ± 2.09	2.11 ± 2.40	1.66 ± 1.15	0.082
Vitamin A (RE/d)	344.02 ± 327.43	271.23 ± 159.48	331.15 ± 482.81	0.306	359.06 ± 329.53	253.80 ± 151.69	333.36 ± 480.74	0.093
Vitamin C (mg/d)	43.14 ± 26.01	35.17 ± 27.92	25.36 ± 21.83	< 0.001	45.16 ± 26.72	34.11 ± 29.12	24.38 ± 17.70	< 0.001
Vitamin B1 (mg/d)	1.30 ± 0.16	1.33 ± 0.18	1.33 ± 0.17	0.391	1.29 ± 0.19	1.34 ± 0.17	1.34 ± 0.15	0.071
Vitamin B2 (mg/d)	0.78 ± 0.17	0.79 ± 0.20	0.86 ± 0.29	0.033	0.80 ± 0.19	0.84 ± 0.23	0.79 ± 0.27	0.352
Vitamin B3 (mg/d)	14.79 ± 1.90	16.13 ± 2.86	18.66 ± 4.42	< 0.001	14.54 ± 2.35	16.12 ± 3.21	18.92 ± 3.65	< 0.001
Vitamin B6 (mg/d)	0.73 ± 0.37	0.65 ± 0.25	1.02 ± 2.60	0.209	0.75 ± 0.39	0.66 ± 0.28	0.99 ± 2.60	0.299
Vitamin B12 (μg/d)	1.60 ± 1.02	1.64 ± 0.84	5.07 ± 18.41	0.035	1.64 ± 1.05	1.78 ± 1.19	4.90 ± 18.42	0.058
Folic acid (μg/d)	107.61 ± 33.63	94.15 ± 28.16	92.51 ± 65.70	0.043	112.12 ± 35.24	97.92 ± 31.85	84.27 ± 61.10	< 0.001

Data are presented as mean ± SD and obtained from analysis of variance (ANOVA).

All values have been adjusted for total energy intake using a residual method.

b Energy intake was not adjusted.

SFA, Saturated Fatty Acids; PUFA, Polyunsaturated Fatty Acids; MUFA, Monounsaturated Fatty Acids; RE, Retinol Equivalents; PRAL, Potential Renal Acid Load; NEAP, Net Endogenous Acid Production.

Beta (*β*) estimates and 95% CIs for biomarkers of inflammation and oxidative stress across tertiles of PRAL and NEAP are presented in Table 3. Serum levels of hs-CRP were positively correlated with a higher PRAL score in patients within the last tertile of PRAL compared to those in the lowest tertile, both before (*β* = 1.67, 95%CI: 0.79, 2.54; P_trend_ < 0.001) and after adjustment for potential confounders (*β* = 1.77, 95%CI: 0.88, 2.65; P_trend_ < 0.001). In the crude model, positive associations were observed between PRAL values and serum concentrations of sICAM-1 (*β* = 80.33, 95%CI: 10.78, 149.88; P_trend_ = 0.024), sVCAM-1 (*β* = 189.93, 95%CI: 73.14, 306.73; P_trend_ = 0.002), and sE-selectin (*β* = 6.12, 95% CI: 1.48, 10.76; P_trend_ = 0.010) for individuals in the third tertile of PRAL compared to those in the first tertile of PRAL. These associations persisted after controlling for age, sex, energy intake, dialysis adequacy, dialysis vintage, serum potassium, serum calcium, serum phosphorous, serum urea, albumin, and BMI. In the crude model, serum levels of hs-CRP (*β* = 1.64, 95%CI: 0.77, 2.51; P_trend_ < 0.001) and sICAM-1 (*β* = 91.56, 95%CI: 22.02, 161.11; P_trend_ = 0.010) tended to be positively associated with NEAP scores in participants of the last tertile of NEAP compared to the first tertile of NEAP. These findings remained significant in the fully adjusted models. A non-significant positive association was found between NEAP and MDA concentrations in the crude model (*β* = 0.28, 95%CI: −0.04, 0.60; P_trend_ = 0.087). However, the association became significant after adjusting for age, sex, energy intake, dialysis adequacy, dialysis vintage, serum potassium, serum calcium, serum phosphorous, serum urea, albumin, and BMI (*β* = 0.35, 95%CI: 0.005, 0.71; P_trend_ = 0.048). No further correlations were seen across PRAL or NEAP tertiles and other variables in the crude and fully adjusted models (Table 3).

**Table 3:** Beta (*β*) and 95% confidence intervals for biomarkers of inflammation and oxidative stress across tertiles of dietary PRAL and NEAP.

	Tertiles of dietary PRAL				Tertiles of dietary NEAP			
	T1	T2	T3	*P* trend	T1	T2	T3	*P* trend
*hs-CRP (mg/L)*								
Crude	Ref	1.03 (0.15, 1.90)	1.67 (0.79, 2.54)	< 0.001	Ref	0.09 (−0.77, 0.96)	1.64 (0.77, 2.51)	< 0.001
Model 1	Ref	1.16 (0.29, 2.09)	1.79 (0.91, 2.67)	< 0.001	Ref	0.10 (−75, 0.96)	1.59 (0.72, 2.45)	< 0.001
Model 2	Ref	1.10 (0.21, 1.99)	1.76 (0.88, 2.65)	< 0.001	Ref	0.09 (−0.80, 0.99)	1.33 (0.44, 2.21)	0.003
Model 3	Ref	1.12 (0.23, 2.01)	1.77 (0.88, 2.65)	< 0.001	Ref	0.10 (−0.79, 1.00)	1.34 (0.46, 2.22)	0.003
*sICAM-1 (ng/mL)*								
Crude	Ref	77.89 (8.34, 147.44)	80.33 (10.78, 149.88)	0.024	Ref	50.55 (−18.98, 120.10)	91.56 (22.02, 161.11)	0.01
Model 1	Ref	79.72 (9.77, 149.67)	80.17 (8.88, 151.47)	0.028	Ref	50.49 (−19.28, 120.27)	90.62 (20.96, 160.28)	0.011
Model 2	Ref	67.51 (−6.38, 141.40)	83.64 (10.26, 157.03)	0.027	Ref	27.84 (−45.78, 101.46)	90.82 (18.46, 163.19)	0.014
Model 3	Ref	63.28 (−10.16, 136.73)	83.21 (10.39, 156.04)	0.026	Ref	26.15 (−46.93, 99.23)	88.83 (16.99, 160.67)	0.015
*sVCAM-1 (ng/mL)*								
Crude	Ref	179.54 (62.75, 296.33)	189.93 (73.14, 306.73)	0.002	Ref	58.75 (−59.84, 177.36)	115.55 (−3.05, 234.15)	0.056
Model 1	Ref	177.35 (60.84, 293.85)	202.67 (83.92, 321.42)	< 0.001	Ref	56.44 (−61.62, 174.51)	119.98 (2.11, 237.85)	0.046
Model 2	Ref	154.96 (33.91, 276.01)	195.01 (74.80, 315.23)	0.002	Ref	31.49 (−91.06, 154.04)	117.33 (−3.11, 237.79)	0.055
Model 3	Ref	151.21 (30.22, 272.19)	194.63 (74.68, 314.58)	0.002	Ref	29.93 (−92.36, 152.24)	115.50 (−4.71, 235.73)	0.059
*sE-selectin (ng/mL)*								
Crude	Ref	3.72 (−0.91, 8.36)	6.12 (1.48, 10.76)	0.01	Ref	2.60 (−2.07, 7.28)	3.10 (−1.57, 7.77)	0.194
Model 1	Ref	2.90 (−1.65, 7.45)	5.43 (0.78, 10.07)	0.022	Ref	2.20 (−2.36, 6.77)	3.27 (−1.28, 7.84)	0.16
Model 2	Ref	2.09 (−2.78, 6.97)	6.65 (1.80, 11.50)	0.007	Ref	1.79 (−3.12, 6.72)	3.60 (−1.24, 8.44)	0.145
Model 3	Ref	2.13 (−2.75, 7.02)	6.66 (1.81, 11.50)	0.007	Ref	1.81 (−3.11, 6.74)	3.62 (−1.21, 8.46)	0.142
*MDA (μmol/L)*								
Crude	Ref	0.19 (−0.12, 0.52)	0.13 (−0.18, 0.46)	0.408	Ref	0.14 (−0.18, 0.46)	0.28 (−0.04, 0.60)	0.087
Model 1	Ref	0.26 (−0.05, 0.58)	0.24 (−0.07, 0.57)	0.137	Ref	0.18 (−0.13, 0.50)	0.29 (−0.02, 0.61)	0.07
Model 2	Ref	0.33 (−0.02, 0.69)	0.21 (−0.14, 0.57)	0.252	Ref	0.23 (−0.12, 0.59)	0.35 (0.005, 0.71)	0.048
Model 3	Ref	0.33 (−0.02, 0.69)	0.21 (−0.14, 0.57)	0.252	Ref	0.23 (−0.12, 0.59)	0.35 (0.005, 0.71)	0.048
*NO (μmol/L)*								
Crude	Ref	1.59 (−1.24, 4.43)	−0.74 (−3.57, 2.08)	0.6	Ref	0.90 (−1.93, 3.74)	−0.25 (−3.09, 2.58)	0.862
Model 1	Ref	1.32 (−1.50, 4.15)	−0.82 (−3.70, 2.04)	0.57	Ref	0.87 (−1.95, 3.69)	−0.08 (−2.90, 2.73)	0.953
Model 2	Ref	0.64 (−2.43, 3.71)	−1.35 (−4.39, 1.69)	0.369	Ref	0.47 (−2.59, 3.54)	0.12 (−2.89, 3.14)	0.938
Model 3	Ref	0.78 (−2.27, 3.83)	−1.34 (−4.36, 1.67)	0.367	Ref	0.54 (−2.50, 3.58)	0.17 (−2.82, 3.17)	0.912
*Endothelin-1 (pg/mL)*								
Crude	Ref	0.07 (−7.33, 7.49)	4.31 (−3.10, 11.72)	0.254	Ref	2.78 (−4.62, 10.19)	5.25 (−2.15, 12.65)	0.165
Model 1	Ref	0.07 (−7.34, 7.48)	5.18 (−2.37, 12.73)	0.18	Ref	3.08 (−4.31, 10.47)	5.78 (−1.60, 13.16)	0.125
Model 2	Ref	−3.02 (−11.05, 4.99)	5.68 (−2.28, 13.65)	0.153	Ref	0.01 (−8.04, 8.06)	5.07 (−2.84, 12.99)	0.206
Model 3	Ref	−3.04 (−11.07, 4.99)	5.68 (−2.28, 13.65)	0.153	Ref	0.02 (−8.03, 8.07)	5.08 (−2.83, 13.00)	0.205

Data are presented as *β* (95% confidence interval) and obtained from linear regression. Crude, Unadjusted.

Model 1: Adjusted for age, sex, and total energy intake.

Model 2: Model 1+ dialysis adequacy, dialysis vintage, serum potassium, serum calcium, serum phosphorous, serum urea, albumin.

Model 3: Model 2 + body mass index.

hs-CRP, High-Sensitivity C Reactive Protein; sICAM, Serum Intercellular Adhesion Molecule; sVCAM, Serum Vascular Cell Adhesion Molecule; MDA, Malondialdehyde; NO, Nitric Oxide; PRAL, Potential Renal Acid Load; NEAP, Net Endogenous Acid Production.

OR and 95% CIs for parameters of malnutrition across tertiles of PRAL and NEAP are summarized in Table 4. Although no significant association was found between PRAL and SGA (OR = 1.35, 95%CI: 0.76, 2.42; P_trend_ = 0.303) in the crude model, this association became marginally significant after controlling for age, sex, energy intake, dialysis adequacy, dialysis vintage, serum potassium, serum calcium, serum phosphorous, and serum urea (OR = 1.98, 95%CI: 0.95, 4.11; P_trend_ = 0.065). Moreover, DMS had a marginally significant positive relationship with PRAL (OR = 1.94, 95%CI: 0.92, 4.05; P_trend_ = 0.077) and NEAP (OR = 2.01, 95%CI: 0.93, 4.31; P_trend_ = 0.072) in fully adjusted models. No significant associations were found between dietary PRAL or NEAP and MIS either before or after adjustment for confounders.

**Table 4:** Odds ratios (OR) and 95% confidence intervals for parameters of malnutrition across tertiles of dietary PRAL and NEAP.

	Tertiles of dietary PRAL				Tertiles of dietary NEAP			
	T1	T2	T3	*P* trend	T1	T2	T3	*P* trend
SGA								
Crude	Ref	1.13 (0.64, 2.01)	1.35 (0.76, 2.42)	0.303	Ref	0.77 (0.44, 1.37)	1.57 (0.87, 2.85)	0.142
Model 1	Ref	1.41 (0.76, 2.63)	1.86 (0.98, 3.53)	0.056	Ref	0.84 (0.46, 1.56)	1.63 (0.86, 3.08)	0.136
Model 2	Ref	1.50 (0.74, 3.06)	1.85 (0.91, 3.77)	0.09	Ref	0.83 (0.41, 1.68)	1.51 (0.73, 3.10)	0.242
Model 3	Ref	1.41 (0.68, 2.88)	1.98 (0.95, 4.11)	0.065	Ref	0.80 (0.39, 1.63)	1.74 (0.82, 3.65)	0.142
DMS								
Crude	Ref	1.01 (0.57, 1.81)	1.33 (0.73, 2.39)	0.343	Ref	0.72 (0.40, 1.28)	1.71 (0.93, 3.14)	0.091
Model 1	Ref	1.18 (0.64, 2.16)	1.65 (0.88, 3.10)	0.115	Ref	0.77 (0.42, 1.40)	1.75 (0.93, 3.10)	0.088
Model 2	Ref	1.36 (0.66, 2.79)	1.78 (0.86, 3.67)	0.117	Ref	0.79 (0.38, 1.60)	1.72 (0.82, 3.61)	0.132
Model 3	Ref	1.28 (0.62, 2.65)	1.94 (0.92, 4.05)	0.077	Ref	0.73 (0.35, 1.52)	2.01 (0.93, 4.31)	0.072
*MIS*								
Crude	Ref	1.06 (0.60, 1.87)	1.20 (0.68, 2.12)	0.52	Ref	0.70 (0.39, 1.23)	1.64 (0.91, 2.93)	0.098
Model 1	Ref	1.22 (0.67, 2.21)	1.50 (0.82, 2.74)	0.185	Ref	0.74 (0.41, 1.34)	1.68 (0.91, 3.07)	0.094
Model 2	Ref	1.43 (0.72, 2.83)	1.55 (0.79, 3.06)	0.207	Ref	0.70 (0.35, 1.40)	1.57 (0.79, 3.12)	0.164
Model 3	Ref	1.37 (0.69, 2.73)	1.59 (0.80, 3.18)	0.183	Ref	0.69 (0.34, 1.37)	1.73 (0.85, 1.50)	0.115

Data are presented as OR (95% confidence interval) and obtained from logistic regression. Crude, Unadjusted.

Model 1: Adjusted for age, sex, and total energy intake.

Model 2: Model 1+ dialysis adequacy, dialysis vintage, serum urea.

Model 3: Model 2 + serum potassium, calcium, phosphorous.

SGA, Subjective Global Assessment; DMS, Dialysis Malnutrition Score; MIS, Malnutrition Inflammation Score.

## Discussion

To the best of our knowledge, this is the first study examining the association between DAL and inflammatory and malnutrition markers in maintenance HD patients. The results of the current study revealed that hs-CRP, sICAM-1, sVCAM-1, and sE-selectin were positively associated with PRAL in subjects with higher scores of PRAL than lower scores of PRAL. These associations remained significant after controlling potential confounders. Additionally, each unit increase in NEAP score was correlated with 1.34, 88.83, and 0.35 times higher serum levels of hs-CRP, sICAM-1, and MDA, respectively. Therefore, this study supports the idea that DAL is independently associated with inflammation. Greater PRAL scores were also correlated with higher odds of SGA and DMS. Moreover, NEAP was linked with a 2.01 times higher chance of DMS.

A potential issue with our findings is the reverse causation hypothesis, meaning the association we studied could exist in the opposite direction. It is worth considering that HD patients should intake 1–1.2 g/kg/day of high biological value proteins such as meat, eggs, and fish (acidic sources) and also consume fewer fruits and vegetables (alkaline foods), which could lead to high DAL scores in these patients; thus, a bidirectional association may exist between DAL and HD outcomes. However, this is only a possibility, and these patients should not be advised to drastically reduce protein intake in order to reduce DAL. Future longitudinal and prospective studies are warranted to investigate the aforementioned correlation.

Previous studies investigated the association between different dietary factors and inflammation and oxidative stress in HD patients (18, 19). A growing body of evidence suggests that DAL is associated with inflammation in other populations (20, 21). Confirming the hypothesis that DAL and inflammation are correlated in HD patients, the present study indicates that patients with the highest DAL scores consumed significantly higher amounts of protein and had greater increases in their levels of inflammatory biomarkers than those with the lowest DAL scores. A limited number of studies have investigated the effects of acidic foods on inflammation and oxidative stress status in HD patients. A clinical trial of 92 HD patients who took 45 grams of whey protein per week observed a significant reduction in hs-CRP and MDA levels after an 8-week intervention (22). Whey protein increases anti-inflammatory and antioxidant activity by scavenging free radicals and improving inflammatory pathways. But, when consumed in high quantities, whey protein can induce inflammatory processes (22). Thus, in the present study, higher protein intakes of participants with the highest DAL scores may also be due to higher intakes of whey protein, which could result in higher level of inflammatory biomarkers. However, a pilot study on 14 chronic HD patients aged 16–71 years showed that red meat snacks (27 grams of protein per day) for 30 days did not affect CRP levels in these patients (23). The discrepancy found with the current study could be related to the differences in the study design, study population, and various potential covariates. Moreover, in a cross-sectional study by Wu et al. conducted on 104 HD patients, there was no association between serum CRP levels and higher intakes of red and processed meats (24). The null findings may be clarified by two potential explanations. One possibility is the mild inflammatory status of HD patients since only 33% of them had CRP concentrations of >5 mg/L. Another potential explanation is their small sample size.

Previous studies found a significant association between inflammation and malnutrition in HD patients (25–27). In this study, a higher predisposition to malnutrition was observed in participants with higher levels of inflammatory biomarkers (T3 vs. T1 of PRAL and NEAP). Accordingly, our findings could also support the positive link between DAL and malnutrition in HD patients. Although these associations were marginally significant, it is possible that larger sample sizes would result in a stronger statistically significant relationship. Despite higher dietary energy and protein intake, subjects in the third tertile of PRAL and NEAP had poor nutritional status when compared to those in the first tertile. Moreover, in this study, lower intakes of total fiber, MUFA, magnesium, vitamin C, and folic acid were observed in participants in the top tertiles of PRAL and NEAP. Taken together, lower intakes of these nutrients could worsen malnutrition status in patients with the highest DAL score. Thus, the contribution of both micro- and macronutrients to nutritional status is important for the management of malnutrition in HD patients. Previous surveys on the association between protein intake and malnutrition status in HD patients reported conflicting results. In a case–control study of 94 stable HD patients compared to 52 healthy individuals, there was no evidence of poor nutritional status despite low protein intake (28). However, Akhlaghi et al. observed a mild-to-moderate malnutrition status in HD patients with lower intakes of protein (29). Moreover, another observational study indicated an inverse association between lower protein intake and malnutrition status in HD patients (30). This inconsistency may be associated with differences in eating habits, statistical methods, and outcome and exposure evaluation measures.

Previous studies demonstrated that the total energy and protein intakes of HD patients were noticeably low compared to recommendations (31, 32). Similarly, in this study, intakes of energy (24.9 ± 10.1 kcal/kg/day) and protein (0.64 ± 0.4 g/kg/day) among participants were noticeably low. It is worth considering that subjects may have underreported energy intake due to the use of a recall-based questionnaire for the assessment of dietary intake. Moreover, we observed that participants in the third tertile of DAL consumed higher amounts of energy compared to the first tertile. There are several explanations regarding this finding. First, diets with higher acid loads are often associated with specific dietary patterns, such as higher intake of animal protein and lower intake of fruits and vegetables. These dietary patterns may contribute to higher overall energy intake. Second, the metabolic breakdown of certain nutrients, such as proteins and sulfur-containing amino acids, can contribute to an acidic environment. Foods that are rich in these nutrients might also be energy-dense, further contributing to a higher energy intake. Additionally, the overall impact of dietary acid load on health is a complex and debated topic within the scientific community. It’s advisable to consider various factors, including overall dietary patterns and lifestyle, rather than focusing solely on a single aspect like dietary acid load.

The exact mechanism for the association between DAL and HD outcomes is still unclear. However, some previous animal and experimental investigations may help to explain our findings. These previous studies observed that acidosis causing tissue damage can induce the expression of inflammatory molecules (e.g., nitric oxide synthases), elevate the activity of inflammatory enzymes (e.g., myeloperoxidase), and increase the level of inflammatory cytokines, like tumor necrosis factor α (TNF-α) (21, 33). Additionally, a higher secretion of pro-inflammatory cytokines, such as interleukin 6, and a lower secretion of anti-inflammatory cytokines, such as interleukin 10, were observed in HD patients with metabolic acidosis (34). Acidosis can affect oxidative stress through incremental free radical formation by H^(+)^-dependent reactions, and iron released from the binding of protein following decrements in pH catalyzes these reactions (35). Furthermore, acidosis diminishes the level of some antioxidants such as glutathione (35, 36) as well as the activity of antioxidant enzymes (37). Previous reports suggest that acidosis is linked with malnutrition in HD patients through an increase in protein catabolism and a decrease in protein synthesis, causing inflammation and insulin resistance, and also diminishing leptin levels (5, 38).

While the current study is novel in exploring the association between DAL and inflammation, oxidative stress, and malnutrition indices in maintenance HD patients as well as considering a comprehensive profile of inflammation and malnutrition concerning DAL, the results should be interpreted with caution due to some limitations. First, due to the cross-sectional design of the present study, no causal relationship can be inferred. Second, the true habitual food consumption of participants was likely underestimated with the 4-day dietary recall. Third, since the present study was conducted on a small sample of HD patients, its generalizability to all patients should be interpreted with caution. Fourth, although several potential confounders were controlled for in our analyzes, the effects of residual confounders cannot be excluded from our results. Finally, the high prevalence of malnutrition and inflammation in this population may lead to an overestimation of the effect size.

We recommend further large-scale studies, especially prospective clinical trials, to validate and explore potential causal relationships. It is crucial to address the following considerations: firstly, employing a 7-day food diary or extending the recall period to assess patients’ typical dietary intake; secondly, accounting for potential confounders such as medications and other dietary factors in the analysis; and thirdly, assessing the body composition and muscle mass of hemodialysis (HD) patients to provide a comprehensive understanding of the observed associations.

## Conclusion

In the current study, high DAL according to PRAL and NEAP scores had a positive significant association with hs-CRP and sICAM-1. Moreover, PRAL scores were positively associated with sVCAM-1 and sE-selectin. NEAP scores also had a positive correlation with MDA. Additionally, high PRAL scores were associated with a higher likelihood of SGA and DMS. A positive correlation was also observed between high NEAP scores and DMS. However, it should not be inferred from our results that HD patients should be recommended to significantly reduce protein intake or increase fruit/vegetable consumption in order to alleviate DAL score and subsequent inflammation and malnutrition. There is a need for longitudinal studies to be conducted to further understand our cross-sectional results and to elucidate potential underlying mechanisms.

## Data availability statement

The data analyzed in this study is subject to the following licenses/restrictions: the data that support the findings of this study are available from the corresponding author upon reasonable request. Requests to access these datasets should be directed to aarab1@bwh.harvard.edu.

## Ethics statement

The studies involving humans were approved by local Ethics Committee of Shahid Beheshti University of Medical Sciences and Health Services. The studies were conducted in accordance with the local legislation and institutional requirements. Written informed consent for participation was not required from the participants or the participants' legal guardians/next of kin in accordance with the national legislation and institutional requirements.

## Author contributions

AB: Writing – original draft, Writing – review & editing. MN: Writing – review & editing. HT: Conceptualization, Data curation, Methodology, Writing – review & editing. AtA: Conceptualization, Methodology, Writing – review & editing. ArA: Formal analysis, Writing – original draft, Writing – review & editing.

## Figures and Tables

**Table:** 

DAL	dietary acid load
HD	hemodialysis
PRAL	potential renal acid load
NEAP	net endogenous acid production
SGA	subjective global assessment
DMS	dialysis malnutrition score
MIS	malnutrition inflammation score
hs-CRP	high-sensitivity C-reactive protein
sICAM-1	soluble intercellular adhesion molecule-1
sVCAM-1	soluble vascular adhesion molecule-1
MDA	malondialdehyde
NO	nitric oxide
ESRD	end-stage renal disease
CVD	cardiovascular disease
MIA	malnutrition inflammation atherosclerosis
MICS	malnutrition inflammation complex syndrome
BMI	body mass index
TIBC	total iron binding capacity
NAE	urinary net acid excretion
TNF	tumor necrosis factor
